# Supplementary material for: Trends in flood losses in Europe over the past 150 years
Source: Nat Commun. 2018 May 29;9:1985. doi: 10.1038/s41467-018-04253-1 (PMC5974183; doi:10.1038/s41467-018-04253-1)
Supplement: Supplementary file 1 — Supplementary Information [file 41467_2018_4253_MOESM1_ESM.pdf]

**Supplemental Information to “Trends in flood losses in Europe over the past 150 years” by Paprotny *et al.***

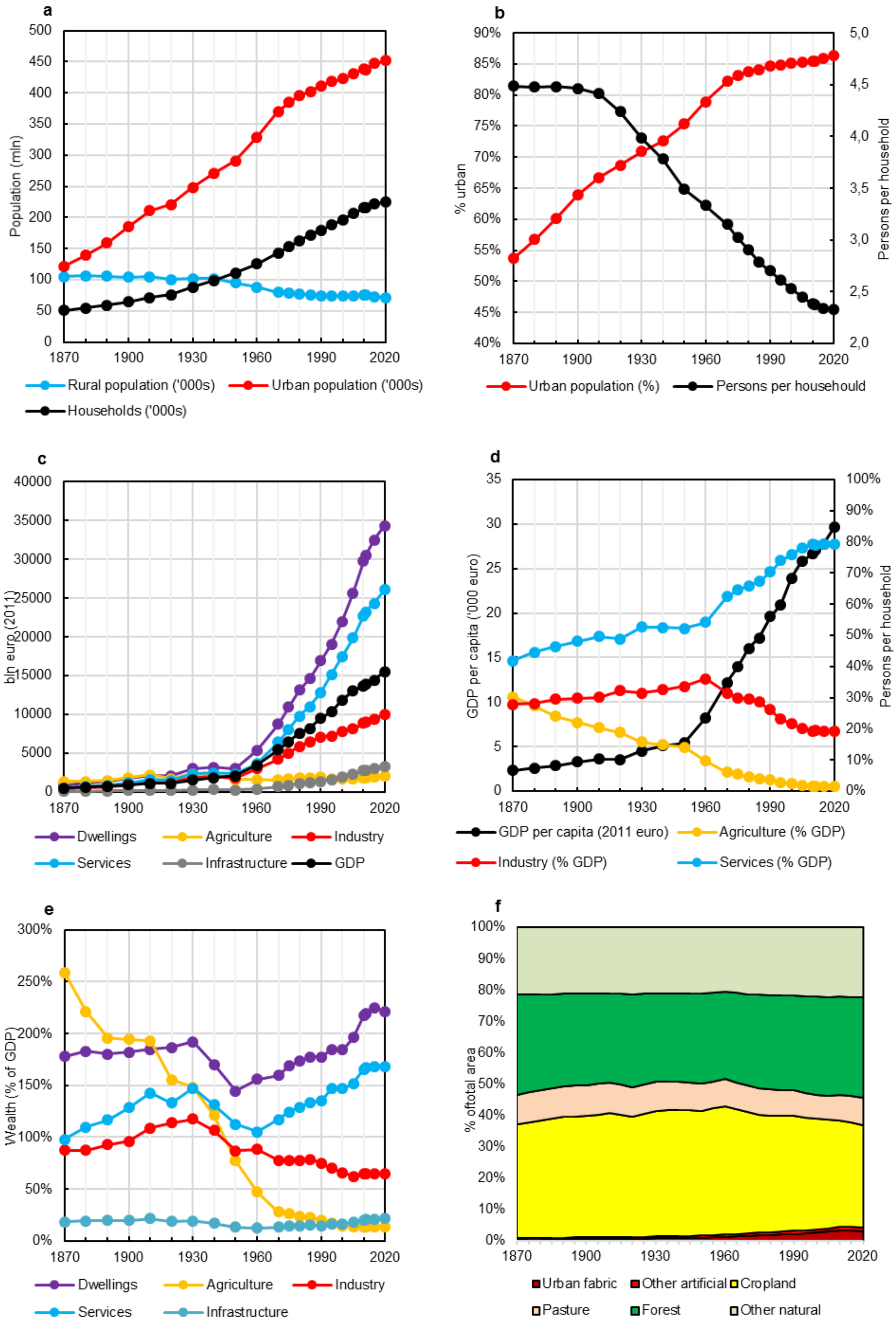


**Supplementary Figure 1. Temporal trends for selected socio-economic variables since 1870: (a-b) population and households; (c) wealth by sector and GPD; (d) GDP structure; (e) wealth by sector relative to GDP; (f) Land use. Graphs include short-term projections through 2020. Aggregated for 37 European countries and territories. Source of data: HANZE database[1].**

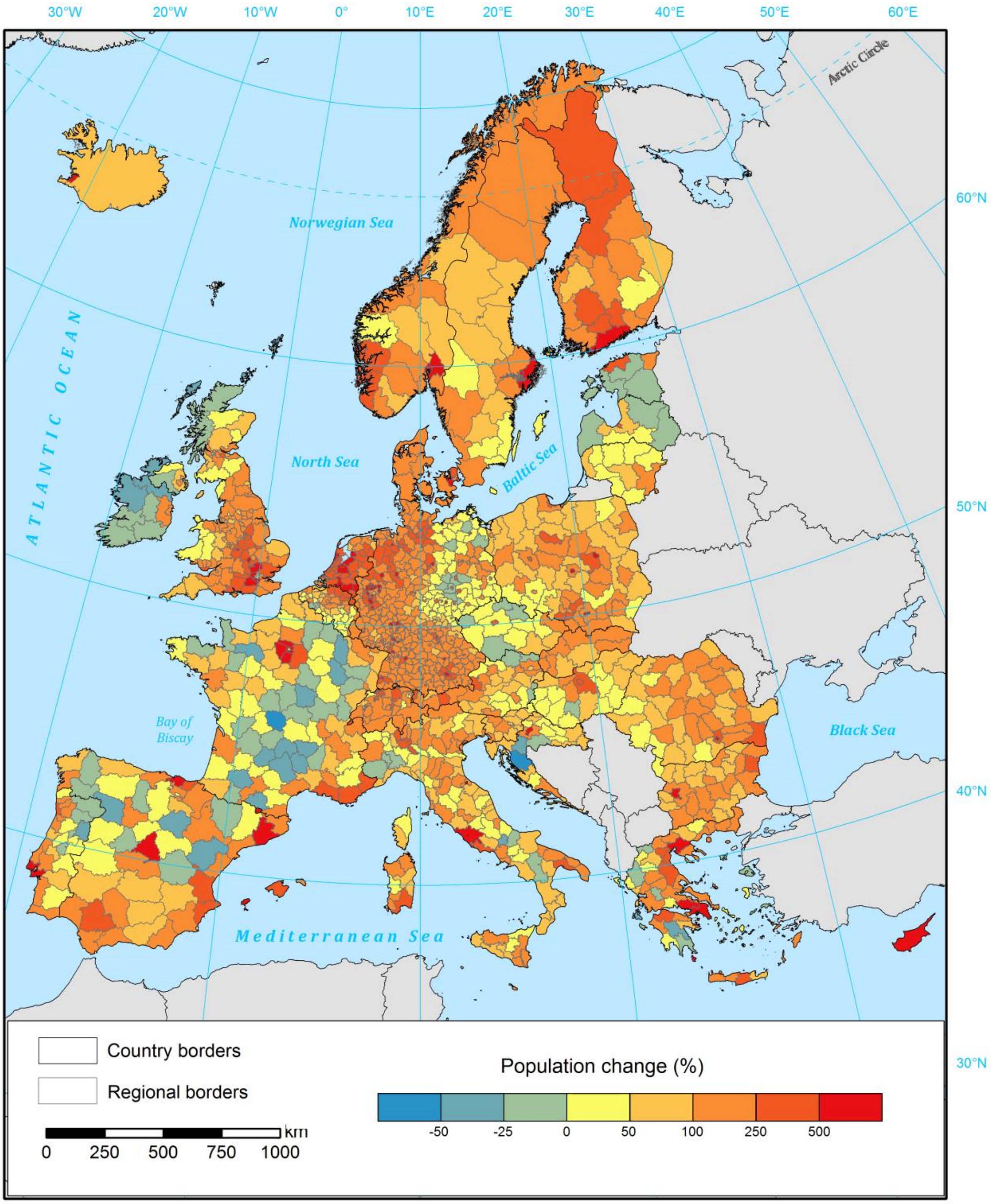


**Supplementary Figure 2. Population change (%) by NUTS3 region from 1870 to 2015. Source of data: HANZE database[1], with background administrative borders from PBL data[2] .**

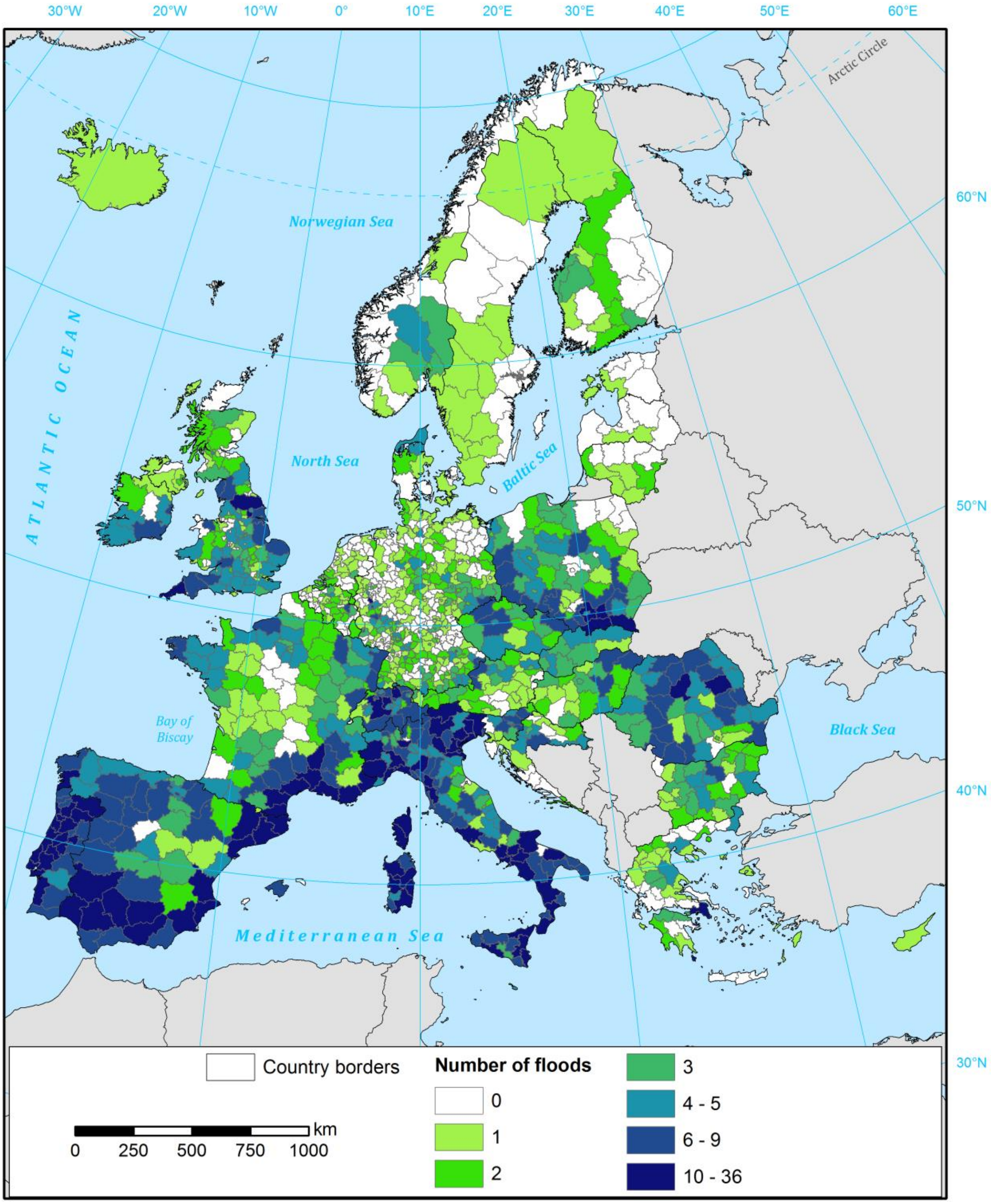


**Supplementary Figure 3. Total number of floods events recorded in HANZE database by NUTS3 region (1870–2016). Source of data: HANZE database[1], with background administrative borders from PBL data[2].**

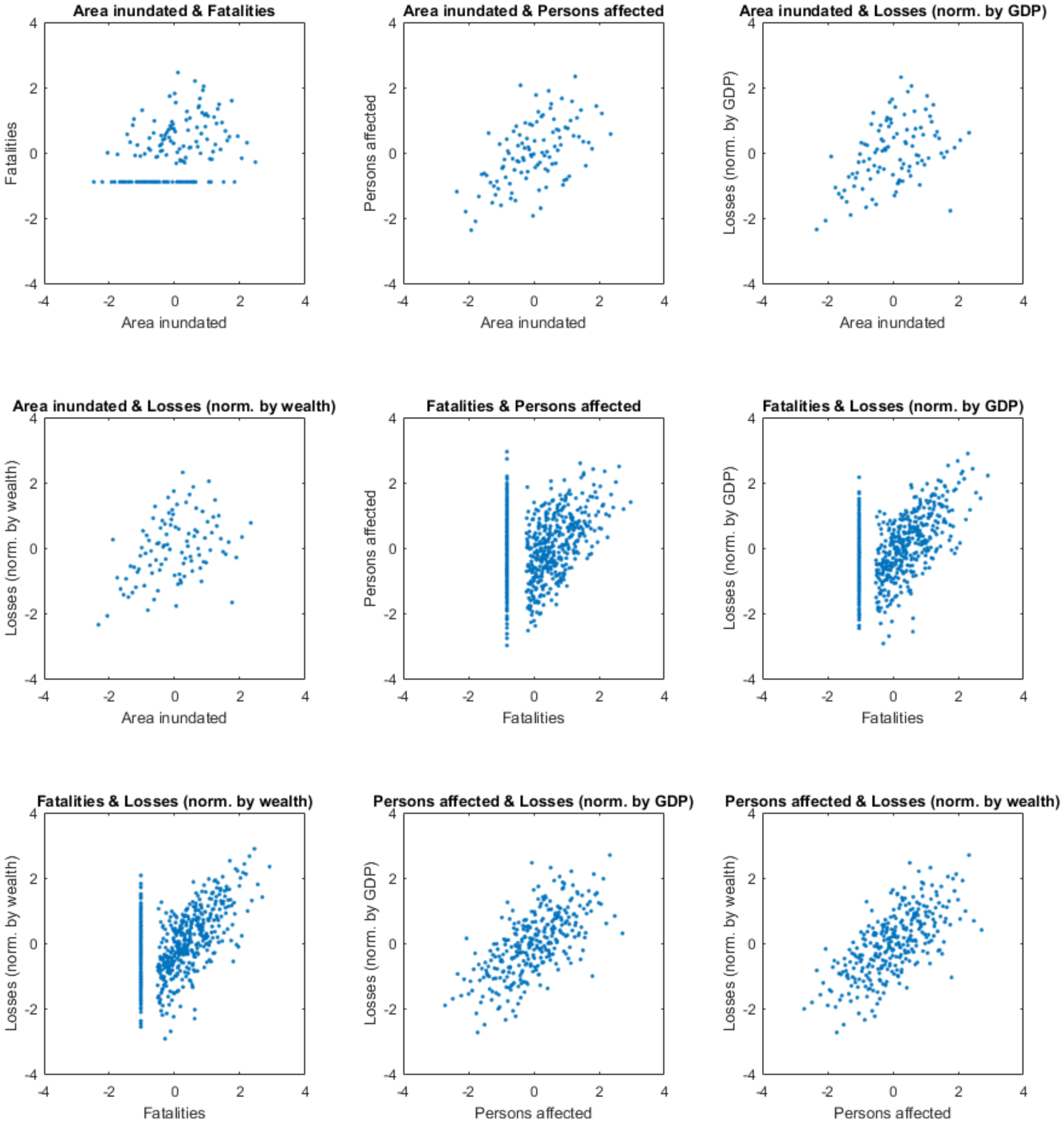


**Supplementary Figure 4. Dependency between pairs of variables (normalized damage statistics relative to potential exposure per flood footprint) transformed to standard normal.**

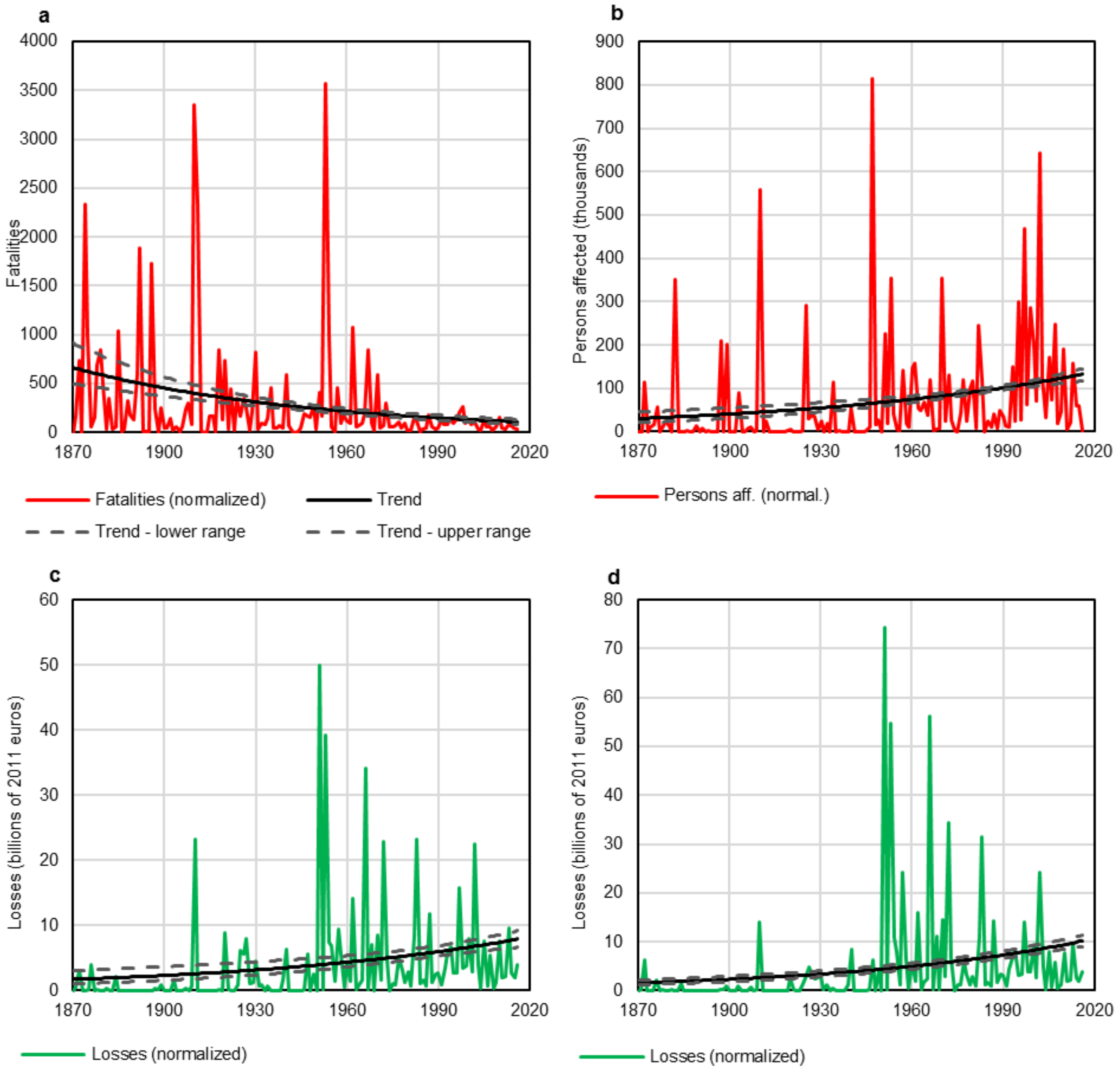


**Supplementary Figure 5. Trends in normalized flood losses with 95% confidence intervals, for (a) fatalities; (b) persons affected; (c) financial value of losses with normalization by GDP and (d) financial value of losses with normalization by wealth.**

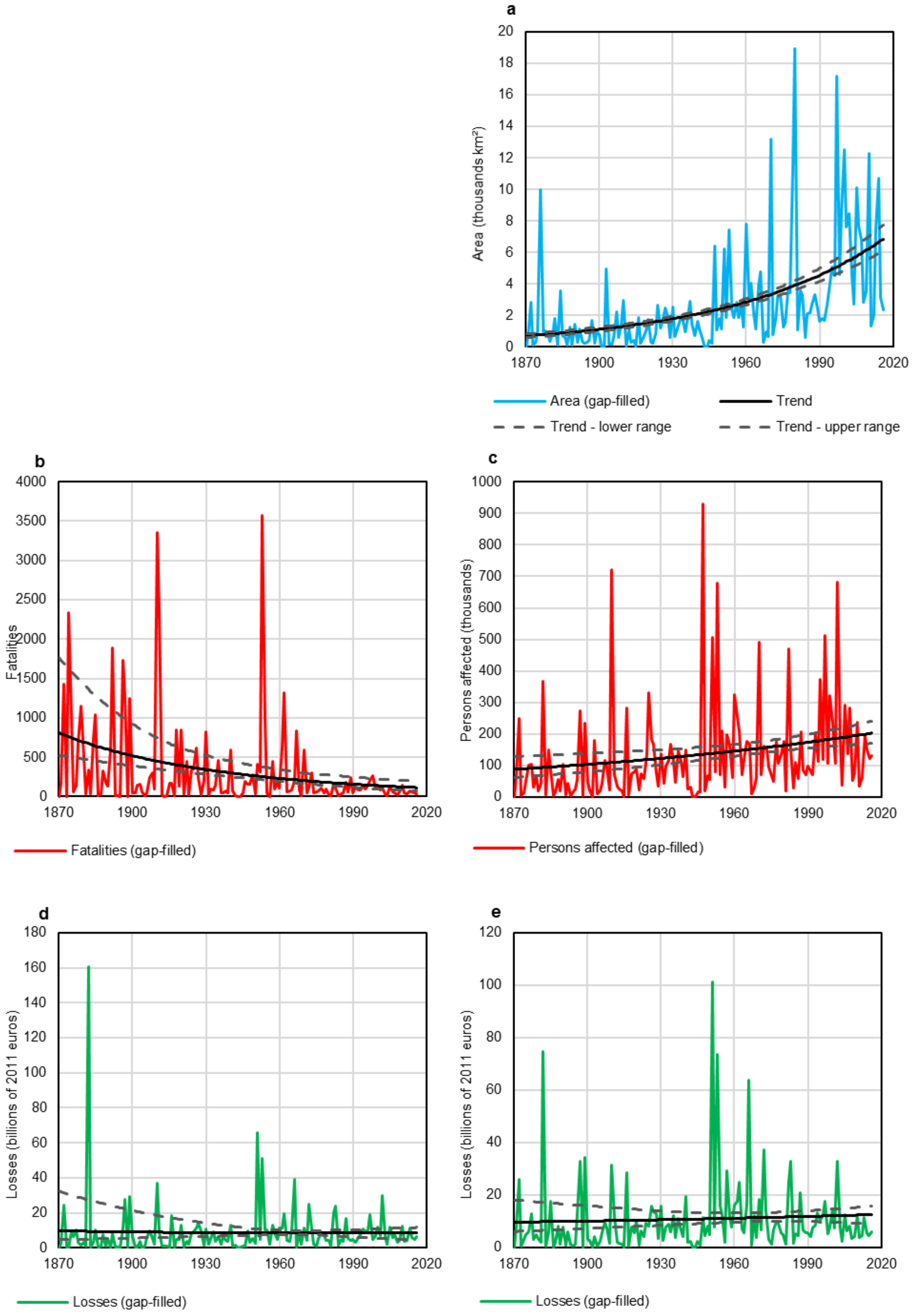


**Supplementary Figure 6. Trends in normalized and gap-filled flood losses with 95% confidence intervals, for (a) area inundated; (b) fatalities; (c) persons affected; (d) financial value of losses with normalization by GDP and (e) financial value of losses with normalization by wealth.**

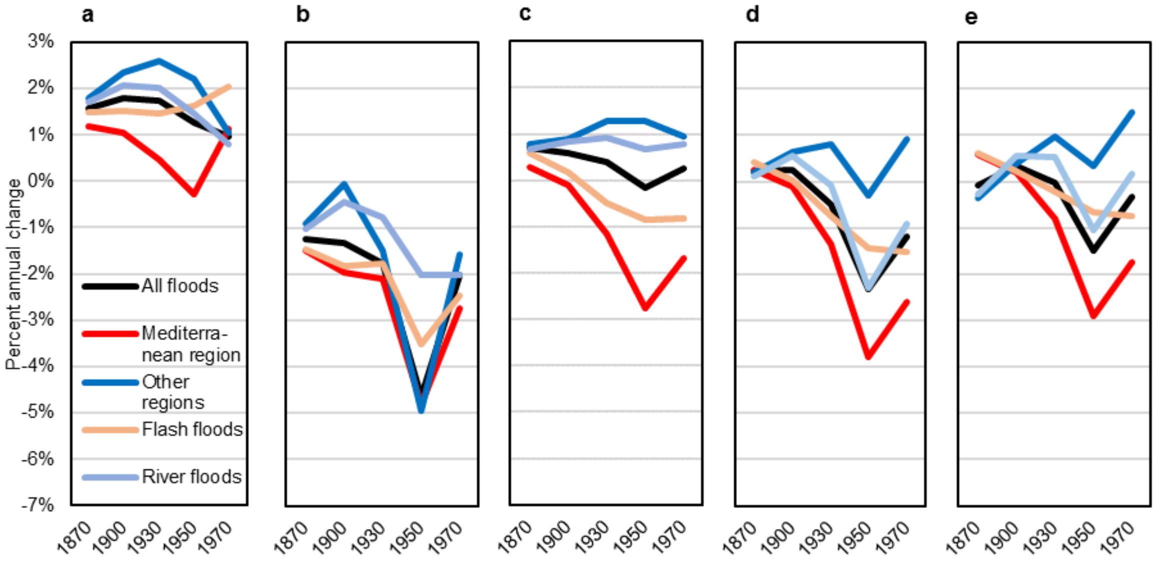


Supplementary Figure 7. Trends in normalized and gap-filled losses for different starting years. Data for all flood events, two subdomains (Mediterranean regions and other parts of Europe) and two types of floods (river and flash), for (a) area inundated; (b) fatalities; (c) persons affected; (d) financial value of losses with normalization by GDP and (e) financial value of losses with normalization by wealth.

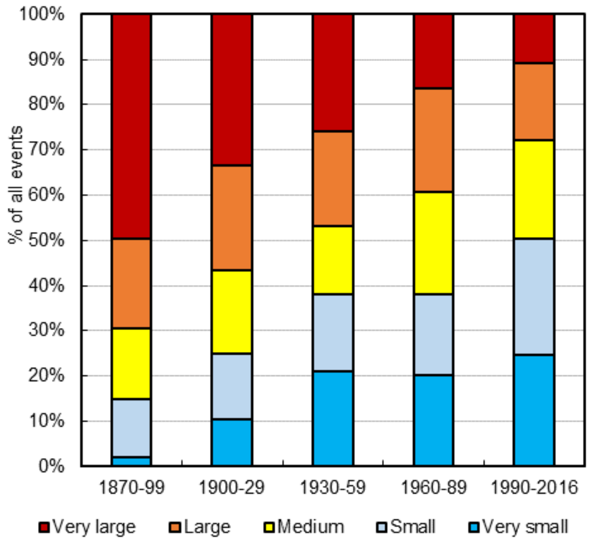


Supplementary Figure 8. Flood events classified by severity per time period.

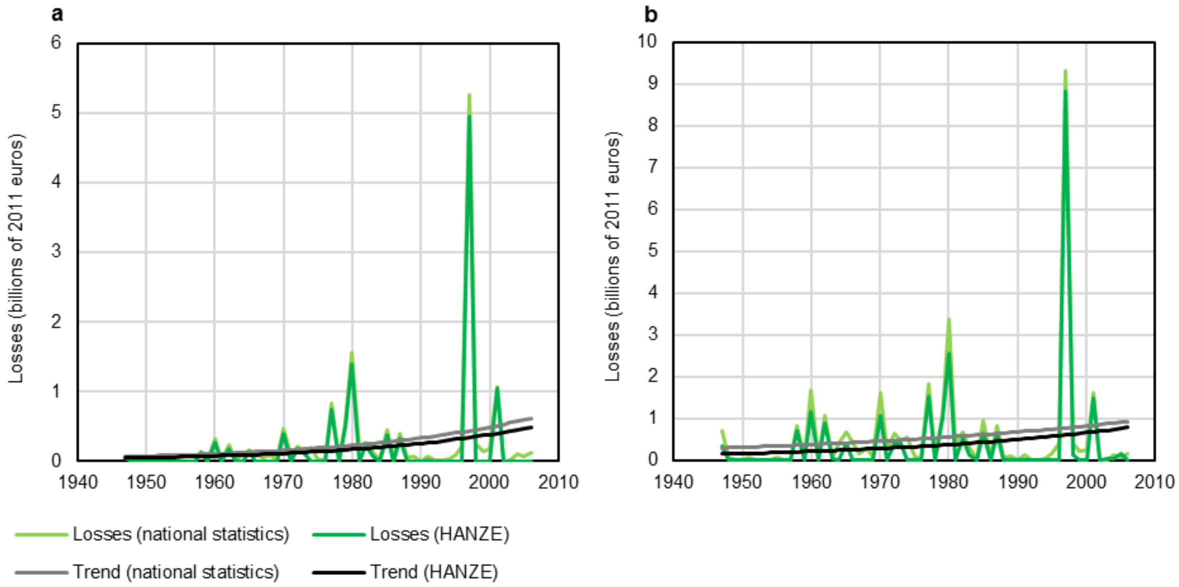


Supplementary Figure 9. Annual financial losses to floods in Poland, 1947–2006, (a) reported and (b) normalized and gap-filled. The trends were calculated using Poisson regression. Source of data: national statistics[2-4] and HANZE database[1].

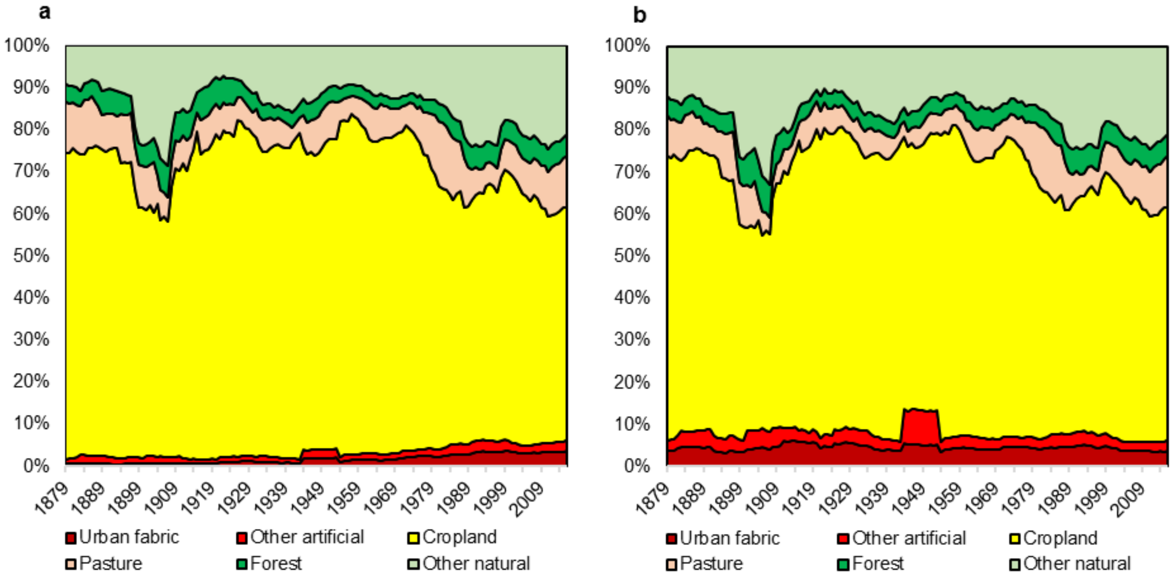


Supplementary Figure 10. Annual land use structure of flood footprints. Graphs are for 10-year moving average (ending on the year indicated), (a) with reconstructed land use at the time of flood events and (b) using 2011 land use. Source of data: HANZE database[1].

Supplementary Table 1. Correlation and best-fitting copulas for pairs of variables (normalized damage statistics).

| Pair of variables | Spearman's rank correlation | Best-fitting copula type |
|---|---|---|
| Area inundated & Fatalities | 0.352 | Frank |
| Area inundated & Persons affected | 0.527 | Clayton |
| Area inundated & Losses (norm. by GDP) | 0.431 | Clayton |
| Area inundated & Losses (norm. by wealth) | 0.376 | Clayton |
| Fatalities & Persons affected | 0.272 | Normal |
| Fatalities & Losses (norm. by GDP) | 0.469 | Gumbel |
| Fatalities & Losses (norm. by wealth) | 0.473 | Gumbel |
| Persons affected & Losses (norm. by GDP) | 0.667 | Frank |
| Persons affected & Losses (norm. by wealth) | 0.677 | Frank |

**Supplementary Table 2. Trends in losses in different aggregations. Data are for reported, normalized and gap-filled annual losses during five periods in the historical record.**

## (a) Mediterranean countries (Cyprus, Greece, Italy, Malta, Portugal, Spain)

| Starting year | Reported | | | | | Normalized | | | | Normalized and gap-filled | | | | |
|---|---|---|---|---|---|---|---|---|---|---|---|---|---|---|
| | Events | Area | Fatalities | Affected | Losses | Fatalities | Affected | Losses [1] | Losses [2] | Area | Fatalities | Affected | Losses [1] | Losses [2] |
| 1870 | *1.2 | *1.6 | -0.5 | *1.6 | *2.3 | *-1.5 | *1.2 | 1.1 | 1.2 | *1.2 | *-1.5 | 0.3 | 0.3 | 0.6 |
| 1900 | *0.8 | *2.6 | -0.1 | *1.6 | *1.9 | *-2.2 | *1.3 | 0.7 | 0.8 | *1.0 | *-2.0 | -0.1 | -0.1 | 0.2 |
| 1930 | 0.2 | 1.9 | -1.0 | 0.6 | 0.9 | -2.3 | 0.1 | -1.0 | -0.8 | 0.5 | *-2.1 | -1.2 | *-1.3 | -0.8 |
| 1950 | *-0.6 | 1.4 | *-3.1 | -1.5 | -1.1 | *-4.5 | -2.2 | *-4.7 | -4.3 | -0.3 | *-4.7 | *-2.7 | *-3.8 | *-2.9 |
| 1970 | 0.3 | 6.8 | -2.2 | -1.7 | -3.1 | -2.4 | -2.5 | -6.0 | -5.3 | 1.1 | -2.7 | -1.7 | -2.6 | -1.7 |

[1] normalized by wealth, [2] normalized by GDP, * significant at α = 0.05.

## (b) Non-Mediterranean countries (other than above)

| Starting year | Reported | | | | | Normalized | | | | Normalized and gap-filled | | | | |
|---|---|---|---|---|---|---|---|---|---|---|---|---|---|---|
| | Events | Area | Fatalities | Affected | Losses | Fatalities | Affected | Losses [1] | Losses [2] | Area | Fatalities | Affected | Losses [1] | Losses [2] |
| 1870 | *2.1 | *1.4 | -0.2 | *2.1 | *3.7 | -0.7 | *1.0 | *1.5 | 0.9 | *1.8 | -0.9 | *0.8 | 0.2 | -0.3 |
| 1900 | *2.5 | *2.0 | 0.6 | *2.1 | *3.6 | 0.1 | 1.1 | *1.2 | 0.4 | *2.3 | 0.0 | *0.9 | 0.6 | 0.4 |
| 1930 | *3.0 | 1.6 | -0.8 | *2.1 | *3.7 | -1.5 | 1.4 | 0.9 | 0.8 | *2.6 | -1.5 | *1.3 | 0.8 | 1.0 |
| 1950 | *3.0 | 0.5 | *-3.5 | *2.6 | *3.2 | *-5.0 | 2.1 | -0.5 | -0.1 | *2.2 | -5.0 | 1.3 | -0.3 | 0.3 |
| 1970 | *2.5 | -2.0 | -1.4 | 2.0 | *4.0 | -1.6 | 1.8 | 1.7 | 2.3 | 1.0 | -1.6 | 1.0 | 0.9 | 1.5 |

[1] normalized by wealth, [2] normalized by GDP, * significant at α = 0.05.

## (c) Flash floods

| Starting year | Reported | | | | | Normalized | | | | Normalized and gap-filled | | | | |
|---|---|---|---|---|---|---|---|---|---|---|---|---|---|---|
| | Events | Area | Fatalities | Affected | Losses | Fatalities | Affected | Losses [1] | Losses [2] | Area | Fatalities | Affected | Losses [1] | Losses [2] |
| 1870 | *1.4 | *2.9 | -0.7 | *2.2 | *3.1 | *-1.6 | *1.7 | 1.5 | 1.5 | *1.5 | *-1.5 | 0.6 | 0.4 | *0.6 |
| 1900 | *1.3 | 2.7 | 0.2 | *2.5 | *2.9 | *-2.1 | *2.0 | 1.3 | 1.1 | *1.5 | *-1.8 | 0.2 | 0.0 | 0.2 |
| 1930 | *0.9 | 2.3 | -0.5 | 1.8 | *2.3 | -1.9 | 1.2 | 0.2 | 0.3 | *1.4 | -1.8 | -0.5 | -0.8 | -0.2 |
| 1950 | *0.6 | 2.2 | -2.2 | 0.6 | 1.2 | -3.2 | 0.2 | -1.3 | -0.9 | *1.6 | *-3.5 | -0.9 | -1.4 | -0.7 |
| 1970 | *0.9 | 2.8 | -1.7 | -0.8 | -0.8 | -2.1 | -1.3 | -3.0 | -2.3 | *2.0 | -2.5 | -0.8 | -1.5 | -0.8 |

[1] normalized by wealth, [2] normalized by GDP, * significant at α = 0.05.

## (d) River floods

| Starting year | Reported | | | | | Normalized | | | | Normalized and gap-filled | | | | |
|---|---|---|---|---|---|---|---|---|---|---|---|---|---|---|
| | Events | Area | Fatalities | Affected | Losses | Fatalities | Affected | Losses [1] | Losses [2] | Area | Fatalities | Affected | Losses [1] | Losses [2] |
| 1870 | *1.7 | *1.4 | 0.0 | *2.0 | *3.5 | -0.8 | *0.9 | *1.3 | 0.9 | *1.7 | -1.0 | *0.7 | 0.1 | -0.3 |
| 1900 | *1.9 | *2.1 | 0.7 | *2.1 | *3.4 | -0.3 | 1.1 | 1.0 | 0.5 | *2.1 | -0.4 | 0.8 | 0.6 | 0.6 |
| 1930 | *1.9 | 1.7 | 0.1 | *2.0 | *3.4 | -0.8 | 1.3 | 0.2 | 0.5 | *2.0 | -0.8 | 0.9 | -0.1 | 0.5 |
| 1950 | *1.6 | 0.8 | -0.4 | 2.2 | *2.7 | -1.9 | 1.7 | -2.0 | -1.2 | *1.5 | -2.0 | 0.7 | -2.3 | *-1.1 |
| 1970 | *2.1 | -1.7 | -2.1 | 1.7 | 2.3 | -2.0 | 1.4 | -1.1 | 0.0 | 0.8 | -2.0 | 0.8 | -0.9 | 0.2 |

[1] normalized by wealth, [2] normalized by GDP, * significant at α = 0.05.

## (e) River, coastal and compound floods

| Starting year | Reported | | | | | Normalized | | | | Normalized and gap-filled | | | | |
|---|---|---|---|---|---|---|---|---|---|---|---|---|---|---|
| | Events | Area | Fatalities | Affected | Losses | Fatalities | Affected | Losses [1] | Losses [2] | Area | Fatalities | Affected | Losses [1] | Losses [2] |
| 1870 | *1.7 | *1.4 | 0.1 | *1.9 | *3.0 | -0.5 | *0.9 | *1.2 | 0.9 | *1.6 | -0.7 | 0.6 | 0.1 | -0.2 |
| 1900 | *1.8 | 2.0 | 0.3 | *1.9 | 2.8 | -0.4 | 1.0 | 0.8 | 0.4 | *1.9 | -0.4 | 0.6 | 0.3 | 0.3 |
| 1930 | *1.8 | 1.6 | -1.2 | *1.7 | 2.4 | -1.9 | 1.0 | -0.4 | -0.1 | *1.8 | -1.9 | 0.7 | -0.5 | 0.0 |
| 1950 | *1.4 | 0.6 | *-4.5 | 1.6 | 1.3 | *-6.4 | 0.9 | -3.3 | -2.6 | 1.2 | *-6.4 | 0.0 | *-3.3 | *-2.2 |
| 1970 | *2.1 | -1.6 | -1.8 | 1.6 | 2.4 | -1.7 | 1.3 | -1.0 | 0.1 | 0.7 | -1.7 | 0.7 | -1.0 | 0.0 |

[1] normalized by wealth, [2] normalized by GDP, * significant at α = 0.05.

## (f) Flash floods (under assumption of flood footprints equalling whole area of affected regions)

| Starting year | Normalized | | | |
|---|---|---|---|---|
| | Fatalities | Affected | Losses [1] | Losses [2] |
| 1870 | *-1.9 | *1.3 | 1.4 | 1.5 |
| 1900 | -0.3 | *2.0 | 1.4 | 1.2 |
| 1930 | -1.0 | 1.3 | 0.3 | 0.3 |
| 1950 | -3.0 | 0.0 | -1.4 | -1.1 |
| 1970 | -2.5 | -1.5 | -3.5 | -2.8 |

[1] normalized by wealth, [2] normalized by GDP, * significant at α = 0.05.

**Supplementary Table 3. Flood footprint characteristics and relative losses. The table shows rank correlation between relative losses (reported versus potential) for the 310 major floods in the uppermost quintile as shown in Supplementary Fig. 8.**

| Relative losses | Flood footprint characteristics | | | | | | | | | | |
|---|---|---|---|---|---|---|---|---|---|---|---|
| | GDP per capita | Population density | Land use structure (% share) | | | | Wealth structure (% share) | | | | |
| | | | urban fabric | other artificial | agri-cultural | natural | agri-culture | industry | services | housing | infra-structure |
| Area inundated | -0.18 | 0.07 | 0.11 | 0.07 | -0.06 | 0.06 | 0.01 | 0.10 | 0.16 | -0.08 | -0.22 |
| Fatalities | -0.29 | -0.26 | -0.34 | -0.12 | 0.10 | -0.02 | 0.30 | 0.12 | -0.14 | -0.23 | -0.23 |
| Persons affected | -0.23 | -0.40 | -0.30 | -0.20 | 0.07 | 0.03 | 0.29 | 0.12 | -0.06 | -0.17 | -0.31 |
| Losses (relative to GDP) | -0.41 | -0.30 | -0.31 | -0.24 | 0.19 | -0.07 | 0.38 | 0.18 | -0.03 | -0.26 | -0.28 |
| Losses (relative to wealth) | -0.41 | -0.33 | -0.34 | -0.24 | 0.19 | -0.08 | 0.38 | 0.16 | -0.01 | -0.28 | -0.28 |

## Supplementary references


1. TU Delft, Faculty of Civil Engineering and Geosciences, Department of Hydraulic Engineering. HANZE: Historical Analysis of Natural Hazards in Europe. *TU Delft. Dataset*. https://doi.org/10.4121/collection:HANZE (2017).
2. PBL Netherlands Environmental Assessment Agency. HYDE ftp://ftp.pbl.nl/hyde/ (2015).
3. Główny Komitet Przeciwpowodziowy. Zarys monografii powodzi w Polsce w 40-lecie działalności Głównego Komitetu Powodziowego (Ministerstwo Ochrony Środowiska i Zasobów Naturalnych, 1988).
4. Mikulski, Z. Gospodarka wodna (Wydawnictwo Naukowe PWN, 1998).
5. Central Statistical Office of Poland http://stat.gov.pl/en/ (2017).
